# Consumption of N_2_O by *Flavobacterium azooxidireducens* sp. nov. Isolated from Decomposing Leaf Litter of *Phragmites australis* (Cav.)

**DOI:** 10.3390/microorganisms10112304

**Published:** 2022-11-21

**Authors:** Undine Behrendt, Tobias Spanner, Jürgen Augustin, Dominik H. Zak, Marcus A. Horn, Steffen Kolb, Andreas Ulrich

**Affiliations:** 1 Leibniz Centre for Agricultural Landscape Research (ZALF), Eberswalder Str. 84, D-15374 Müncheberg, Germany; 2 Institute of Microbiology, Leibniz University Hannover, Herrenhäuser Str. 2, D-30419 Hannover, Germany; 3 Institute for Ecoscience, Aarhus University, C.F. Møllersvej, Bygning 1331, 8000 Aarhus, Denmark; 4 Leibniz-Institute of Freshwater Ecology and Inland Fisheries Berlin, Müggelseedamm 301, D-12587 Berlin, Germany

**Keywords:** *Flavobacterium azooxidireducens* sp. nov., phylogenomic analysis, nitrous oxide reduction, Clade II *nosZ*, non-denitrifier

## Abstract

Microorganisms acting as sinks for the greenhouse gas nitrous oxide (N_2_O) are gaining increasing attention in the development of strategies to control N_2_O emissions. Non-denitrifying N_2_O reducers are of particular interest because they can provide a real sink without contributing to N_2_O release. The bacterial strain under investigation (IGB 4-14^T^), isolated in a mesocosm experiment to study the litter decomposition of *Phragmites australis* (Cav.), is such an organism. It carries only a *nos* gene cluster with the sec-dependent Clade II *nosZ* and is able to consume significant amounts of N_2_O under anoxic conditions. However, consumption activity is considerably affected by the O_2_ level. The reduction of N_2_O was not associated with cell growth, suggesting that no energy is conserved by anaerobic respiration. Therefore, the N_2_O consumption of strain IGB 4-14^T^ rather serves as an electron sink for metabolism to sustain viability during transient anoxia and/or to detoxify high N_2_O concentrations. Phylogenetic analysis of 16S rRNA gene similarity revealed that the strain belongs to the genus *Flavobacterium*. It shares a high similarity in the *nos* gene cluster composition and the amino acid similarity of the *nosZ* gene with various type strains of the genus. However, phylogenomic analysis and comparison of overall genome relatedness indices clearly demonstrated a novel species status of strain IGB 4-14^T^, with *Flavobacterium lacus* being the most closely related species. Various phenotypic differences supported a demarcation from this species. Based on these results, we proposed a novel species *Flavobacterium azooxidireducens* sp. nov. (type strain IGB 4-14^T^ = LMG 29709^T^ = DSM 103580^T^).

## 1. Introduction

Mitigating global climate change requires a comprehensive understanding of the mechanisms that produce and consume greenhouse gases. Various biotic and abiotic processes are involved in the release of nitrous oxide (N_2_O), the potent greenhouse gas and the dominant source of stratospheric ozone depletion [1,2]. As part of efforts to develop strategies to control N_2_O emissions, increasing attention is being paid to the reduction of N_2_O by microorganisms. The most important biologically catalysed N_2_O reduction is conducted by the copper cluster-containing enzyme N_2_O reductase NosZ [3]. In the past, N_2_O consumption has been attributed primarily to canonical denitrifying bacteria. However, genomic-based studies identified “atypical” NosZ coding genes (Clade II *nosZ*) [4] that are often more abundant in many biomes than the well-studied “typical” *nosZ* genes (Clade I *nosZ*) [5], which denitrifying bacteria typical possess. Furthermore, diversity is generally higher for Clade II *nosZ* than for Clade I, which is reflected by a larger taxonomic range of Clade II *nosZ* among sequenced genomes [2].

Organisms containing Clade II *nosZ* genes possess divergent *nos* gene clusters with genes that are evolutionarily distinct from the typical *nos* genes of denitrifiers [4,6,7]. The *nos* gene clusters have a gene upstream *nosZ*, which encodes a transmembrane protein (*nosB*), and are often associated with *c*- and *b*-type cytochromes, as well as iron–sulfur protein encoding genes. In contrast, the *nosR* and *nosX* genes, reported as essential to maintain the activity of Clade I NosZ, are absent in Clade II *nos* gene clusters. Another difference lies in the signal peptide for NosZ export across the cytoplasmic membrane [3,6]. Nearly all Clade II *nosZ* possess Sec-dependent (secretory pathway) signal peptides to transport the enzyme in unfolded form, whereas the Clade I NosZ signal peptide is recognised by the twin-arginine translocation system (Tat), where it is transported in folded form. Overall, the question of how far these obvious genomic differences between organisms with Clade I and II *nosZ* are translated into physiological differences that may influence niche differentiation within and between the individual clades cannot yet be clearly answered and requires further studies [2]. There is an open debate about whether N_2_O-reducing bacteria of Clade II have an inherently higher N_2_O affinity than their Clade I counterparts [6,8,9,10,11,12]. However, the clade type does not fully explain the different N_2_O affinities, indicating that the physiological properties of N_2_O-reducing bacteria differ at the species and strain levels. However, slightly more than half of Clade II *nosZ* organisms are apparently non-denitrifying N_2_O reducers and therefore have the potential to be a sink without contributing to N_2_O release [2,13].

Members of the genus *Flavobacterium* are widespread in the environment and occur in very diverse habitats—from marine and fresh waters, sediments and soils, various plant compartments, food, and food processing plants to clinical environments [14,15,16]. Although the reduction of nitrate (NO_3_^−^) to nitrite (NO_2_^−^) was known to occur in many species of the genus, denitrification was first described for the species *Flavobacterium denitrificans* in 2005 [17,18]. However, microbial communities in N_2_O biofiltration systems included *Flavobacterium* spp. as one of the most abundant Clade II *nosZ*-carrying organisms [19,20]. Analysis of enrichment cultures of activated sludge samples in a continuous N_2_/N_2_O stream revealed also a dominance of *Flavobacterium* spp. among both *nosZ* gene and transcript pools [21]. These results demonstrate a high potential of flavobacteria to serve as an N_2_O sink in natural or man-made environments. Screening available genomes of type strains for genes encoding enzymes for the reduction of different nitrogen species [22], we found a large diversity of strains harboring such genes, suggesting that these traits are widespread in *Flavobacterium* species. In addition, a high degree of modularity was found in the gene inventory, which also indicates functional differences.

Accompanying a mesocosm experiment to study the litter decomposition of *Phragmites australis* (Cav.) under different redox conditions, the NO_3_^−^/NO_2_^−^ reducing bacterial community was investigated in relation to N_2_O and ammonia flux measurements. In this context, a group of isolates was obtained that were assigned to the genus *Flavobacterium*. This group was interesting both from a taxonomic point of view and with regard to functional traits related to N-transformation processes. A primarily analysis of 16S rRNA phylogeny revealed a novel species status and a screening for reduction of different nitrogen species demonstrated the ability to consume N_2_O. Therefore, in this study the exact taxonomic position of this group was investigated by a polyphasic approach. Furthermore, physiological characteristics and the gene inventory were studied to determine the potential to participate in N-transformation processes.

## 2. Materials and Methods

### 2.1. Isolation and Primariy Classification

The isolates studied originate from a mesocosm experiment designed to investigate the litter decomposition of *Phragmites australis* (Cav.) under different redox conditions. Leaves were collected from Phragmites growing in a rewetted fen (Germany, 53°52′33.4′′ N, 12°53′20.7′′ E) placed in stainless steel litterbags (1 mm mesh size) and processed as described by Reuter, et al. [23]. The litterbags were transferred into 5 L Duran wide neck bottles with four port caps and incubated in artificial fen water medium (4.5 L). Therefore, organic-free, deionized water was supplemented with NaHCO_3_ and fresh detritus mud from the plant-sampling site as an inoculum. Furthermore, 200 mgL^−1^ NO_3_^−^ or SO_4_^2−^ was added as an oxidant in comparison to a control without external oxidants. The mesocosms were incubated under dark conditions in a custom-built flow-through steady-state system which is described in detail by Rillig, et al. [24]. A temperature of 18 °C was maintained in the incubation vessels with the aid of a climate chamber. The headspace of the mesocosms (0.5 L) was continuously flushed with nitrogen (N_2,_ 6 Lh^−1^) using a nitrogen membrane-generator NGM-11-LC/MS (CMC Instruments GmbH, Eschborn, Germany). Gas flux measurements were performed continuously during the whole incubation period according to Rillig, et al. [24].

To investigate the microbial community, leaves of a litter bag (10 g) were immersed in 90 mL of 0.2 M Sörensen sodium phosphate buffer (pH 7) and treated for 2 min at high speed in the Stomacher 400 Circulator (Seward Ltd., Thetford, UK). Serially diluted samples were inoculated onto selective growth medium G3M12, which was optimized for the isolation of NO_3_^−^ reducing bacteria [25]. The medium consisted of a standard mineral base according to Stanier, et al. [26] supplemented with Hutner’s vitamin-free mineral base [27] and a thiamine and complex vitamin solution [25,28]. The C/N ratio was adjusted to 2.5, with ethanol added as a carbon source and KNO_3_ as a nitrogen source. The pH of the medium was corrected to 7.5 after autoclaving and addition of sterile filtered supplements. The agar plates were incubated for 2 weeks at 21 °C in an anoxic chamber [gas composition: 10% carbon dioxide (CO_2_), 5% hydrogen (H_2_), and 85% N_2_]. Forty isolates were randomly picked and purified on ½ strength nutrient agar II containing 15 gL^−1^ agar (½NAII; composition according to Sifin, Berlin, Germany) to classify the isolates according their taxonomic affiliation and functional traits in the nitrogen cycle. Classification of isolates according to their taxonomic background was performed by matrix-assisted laser desorption/ionization time of flight mass spectrometry (MALDI-TOF MS) as described by Ulrich, et al. [29].

### 2.2. Phylogenetic Analysis

The taxonomic classification was confirmed by sequencing the almost complete 16S rRNA gene [30]. The 16S rRNA gene was amplified using primers 8f and 1525r [31] according a protocol described by [32] and sequenced with the internal primers 1492r [31] and 782r [33]. Sequence similarity comparisons were carried out using the EzBiocloud database [34]. For the phylogenetic analysis, an alignment (1488 nt) of the 16S rRNA genes from closely related species was generated using the ClustalW algorithm with MEGA X [35]. A phylogenetic tree was constructed using the maximum-likelihood method based on evolutionary distances of the General Time Reversible model (+G+I) and the neighbour-joining algorithm applying the Kimura 2-parameter model (+G). To investigate a possible clonal origin Ribotyping was performed with restriction endonucleases *Eco*RI, *Pvu*II as described by Behrendt, et al. [36]. Furthermore, ribosomal intergenic spacer regions of isolates were amplified with the primers 1492f and 115r and subsequently sequenced as described by Tokajian, et al. [37].

### 2.3. Genome Sequencing and Bioinformatic Analyses

Strain IGB 4-14^T^ was cultured in nutrient broth II (NBII; Sifin, Germany) for 2 days at 25 °C. The whole genomic DNA was extracted as described by Ulrich et al. [38]. DNA was sequenced using the Pacific Biosciences (PacBio) RS II sequencing platform at Eurofins Genomics (Konstanz, Germany). Sequence reads were de novo assembled using the PacBio hierarchical genome assembly process (HGAP4). The assembly resulted in one contig with an average genome coverage of 209x. The genome sequence was circularized with Circlator ver. 1.5.5 and deposited in the GenBank database under accession no. CP096205.

For the phylogenomic analysis, a tree based on core genome phylogeny [39] was constructed as described by Behrendt et al. [40]. The analysis of 120 bacterial core marker genes resulted in a concatenated amino acid sequence alignment, which was used to calculate a maximum-likelihood tree (LG substitution model with F+G+I) with MEGA X [35]. Digital DNA-DNA hybridization (dDDH) and genomic G+C content were determined on the Type Strain Genome Server (TYGS; [41]). Average nucleotide index (ANI) values were calculated by the OrthoANIu procedure [42]. To support the taxonomic classification, average amino acid identity (AAI) as a further genome relatedness index was calculated using the EzAAI tool [43].

The genome sequences of IGB 4-14^T^ and related species were annotated with Rapid Annotation using Subsystem Technology (RAST) version 2.0 [44,45]. Additional functional and pathway analyses were performed using the BlastKOALA web tool of the KEGG database [46] and the NCBI Prokaryotic Genome Annotation Pipeline [22]. To analyse the *nosZ* gene phylogeny amino acid sequences of type strains representing the genus *Flavobacterium* and reference strains for Clade II *nosZ* genes according to Sanford, et al. [4] were selected. A maximum-likelihood tree (LG substitution model with G+I) was calculated with MEGA X [35].

### 2.4. Phenotypic Characterisation

Phenotypic analyses were performed for strain IGB 4-14^T^ and the reference strain *Flavobacterium lacus* NBRC 109715^T^. Standard methods for morphological and physiological characterization were conducted as described by Behrendt, et al. [47]. Unless otherwise stated, strains were cultivated on ½NAII or in the respective broth (½NBII) at 22 °C. Presence of oxidase was tested on Cytochrome Oxidase Test Strips (Merck). Production of flexirubin-type pigments and Congo red absorption were assessed following the methods of Bernardet, et al. [48]. Oxidation of carbon compounds and resistance to inhibitory chemicals were determined using GEN III MicroPlates (Biolog) according to the manufacturer’s instructions. Results were scored visually after 24 and 48 h. Additional physiological and enzymatic characteristics were determined using the API 20E and API 20NE test strip (bioMérieux) after 48 h.

Analysis of cellular fatty acids was performed at DSMZ GmbH (Braunschweig, Germany) after cultivation on tryptic soy agar (TSA) for 2 days at 20 °C. Cellular fatty acids were converted into fatty acid methyl esters (FAMEs) using minor modifications of the method of Miller [49] and Kuykendall, et al. [50]. The FAMEs were separated by gas chromatography and detected by a flame ionisation detector using Sherlock Microbial Identification System (MIDI, Microbial ID, Newark, DE, USA). Peaks were automatically integrated, and fatty acid identifications and percentages were calculated by standard software (Microbial ID, Library TSBA40, 4.10).

### 2.5. N_2_O Consumption

**Figure 4:**
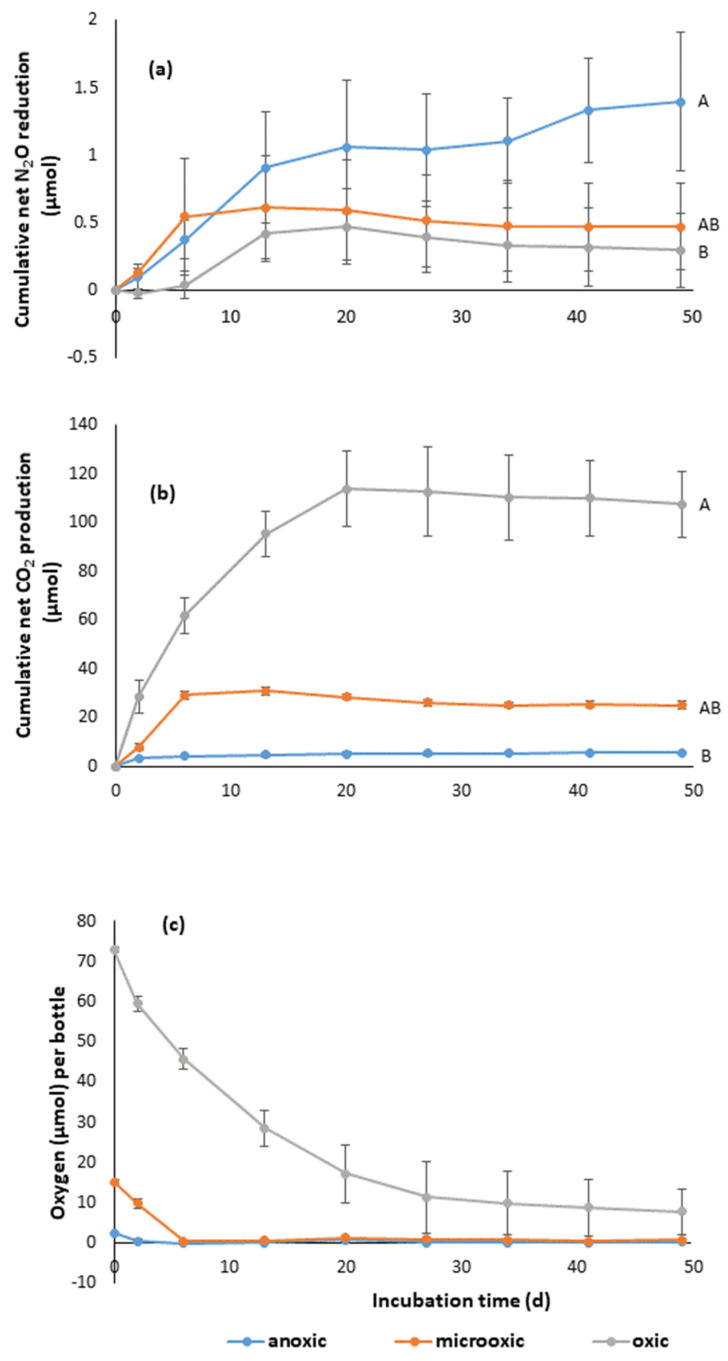
(**a**) Cumulative net N_2_O reduction; (**b**) cumulative net CO_2_ production; and (**c**) O_2_ depletion by strain IGB 4-14^T^ under anoxic, microoxic (4% O_2_) and oxic (20% O_2_) conditions. The plotted values are averages of six repetitions, corrected by the average of three controls. Error bars represent the standard deviations. Different letters indicate significant differences (*p* < 0.05).

Strain IGB 4-14^T^ was cultured under oxic conditions in ½NBII for 48 h at 20 °C. 100 µL of this culture was inoculated into sterile 12 mL flat bottom vials (Labco, Lampeter, UK) containing 5 mL ½NBII broth. To create anoxic or micro-oxic conditions, the headspace was replaced three times with helium. For micro-oxic conditions, sterile air was added to achieve 4% (*v/v* headspace) oxygen (O_2_). Furthermore, N_2_O and nitric oxide (NO) were added into vials of all experiments (oxic, micro-oxic and anoxic) at a final concentration of 0.8% (*v/v* headspace) and 6 ppm (*v/v* headspace), respectively. NO was included as an inducer molecule for transcription of the *nos* genes [51]. To avoid diffusion with the ambient air, an overpressure (0.05 MPa) was created with helium in the anoxic and micro-oxic experiment and with sterile air in the oxic experiment. All tests were carried out in six replicates. For each experiment, a control was performed in triplicate without inoculation. The inoculated and control vials were incubated at 20 °C and sampled for gas analysis according to the time sequence shown in Figure 4. N_2_O, O_2_ and CO_2_ were measured via gas chromatography (7890B GC Agilent Technologies Inc., Santa Clara, CA, USA) equipped with an autosampler (Combi Pal-xt system, CTC analytics, Zwingen, Switzerland). A thermal conductivity detector (TCD, G3440B, Agilent Technologies Inc., Santa Clara, CA, USA) was used for measurement of O_2._ A pulsed discharge helium ionization detector (PDHID, V1D-3-I-HP220, Valco Instruments Company Inc. VICI AG International, Houston, TX, USA) detected CO_2_ and N_2_O. Details of the method are described in Zaman, et al. [52].

Increase in optical density (OD_600_) was used for estimating cell growth. It was measured at the start and the end of the experiment in a microplate with a Tecan plate reader (Infinite M Plex, Tecan Trading AG, Männedorf, Switzerland) and path length correction.

Loss of N_2_O, O_2_ and CO_2_ due to gas sampling was accounted for in the subsequent calculations. Equilibrated headspace concentrations of gases were used to allow comparative assessments of reduction or production of gas. Statistical significance was analyzed with a Kruskal-Wallis-Test and Dunn’s post hoc test of multiple comparisons using rank sums adjusted with the Bonferroni method.

## 3. Results and Discussion

Based on a similarity analysis of the spectral data obtained by MALDI TOF-MS, the isolated strains were classified into taxonomic units at the species level. A group of isolates that could not be assigned to any taxa using the MALDI biotyper reference database originated from the 200 mgL^−1^ NO_3_^−^-supplemented mesocosm. The abundance of this group was 2 × 10^5^ colony forming units per gram of fresh leaf litter. By 16S rRNA gene sequence comparison, the group could be assigned to the genus *Flavobacterium* of the phylum *Bacteroidota*, but a clear species affiliation was still not possible. Analysis of ribosomal intergenic spacer regions of five randomly selected isolates revealed identical sequences. Furthermore, the riboprint patterns showed no differences at the strain level. These results clearly indicated a clonal origin of the isolates. Therefore, only one isolate, strain IGB 4-14^T^, was selected for detailed taxonomic and physiological studies.

### 3.1. Phylogenetic Analysis

**Figure 1:**
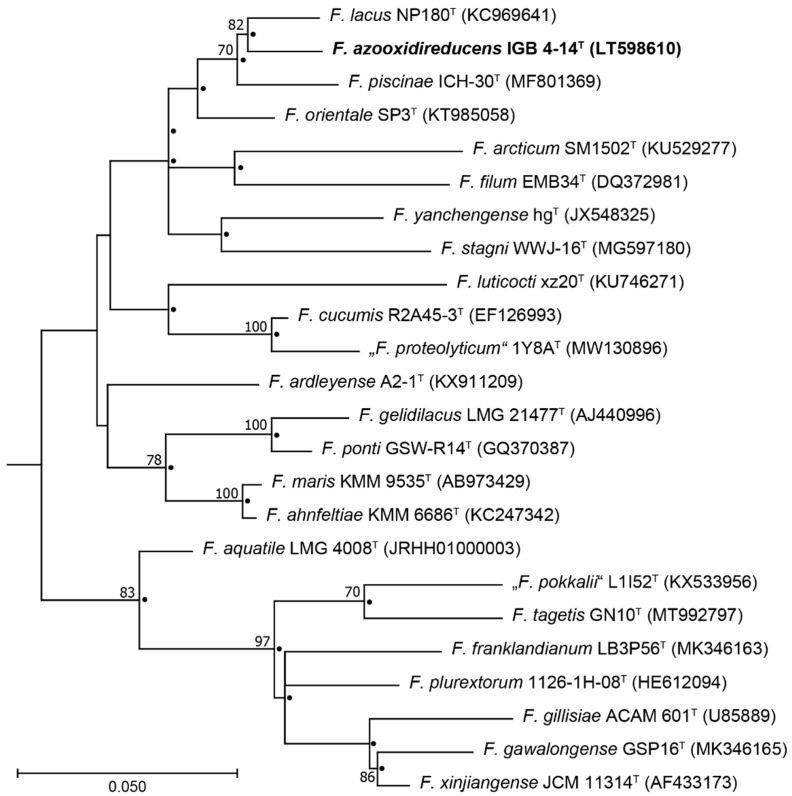
Maximum-likelihood tree of the 16S rRNA gene sequences showing the position of strain IGB 4-14^T^ among phylogenetically related species of genus *Flavobacterium*. Filled circles indicate branches of the tree that were also obtained using neighbour-joining algorithm. The sequence GU166749 of *Lutibacter flavus* IMCC1507^T^ was used as an outgroup. Numbers at branch nodes refer to bootstrap values ≥70%. Bar: substitutions per nucleotide site. Accession numbers (NCBI or IMG database) are indicated in brackets.

Comparative 16S rRNA gene analysis of strain IGB 4-14^T^ revealed the highest sequence similarity of 97.3% to the species *F. lacus* (Table 1). Considering the species boundary of 98.2–99.0% for the 16S rRNA gene sequence similarity proposed by Meier-Kolthoff, et al. [53], a separate species position of the isolated strain is indicated. Furthermore, the high abundance of the isolates indicated an active growth under the tested redox conditions. The phylogenetic tree demonstrated that the isolated strain clearly clusters with *F. lacus* NP180^T^ in both treeing methods applied, which was additionally supported by a high bootstrap value of 82% (Figure 1). Their position in relation to a cluster formed by further species of the genus *Flavobacterium* could also be found by both treeing methods. However, only the position related to the species *Flavobacterium piscinae* was confirmed by a supporting bootstrap value of 70%.

It is known that phylogenetic analyses based on the 16S rRNA gene do not always provide the desired taxonomic resolution depending on the genus studied [54]. Nevertheless, it gives a complete picture regarding the relationships within the genus because genomes are not available for all species. However, the study of evolutionary relationships using orthologous genes of the core genome can be used for a more solid and alternative way for phylogenetic analysis [39]. Therefore, the genome of the strain was sequenced. The assembled genome comprises a single circular contig with a total length of 3,823,204 bp exhibiting 100% completeness. No plasmid is present. The genome encoded 3263 protein-coding sequences and the DNA G+C content calculated from the genome sequence is 33.8%, which is consistent with the description of the genus *Flavobacterium* [18].

**Figure 2:**
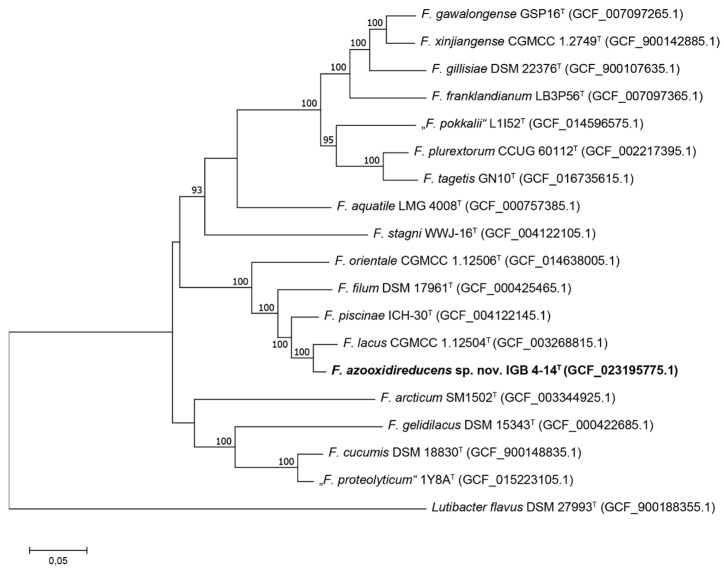
Phylogenomic tree showing the position of strain IGB 4-14^T^ within the genus *Flavobacterium*. The strain *Lutibacter flavus* IMCC1507^T^ was used as an outgroup. The maximum-likelihood tree is based on concatenated 120 core marker proteins. Numbers at branch nodes refer to bootstrap values >70%. Bar: amino acid substitutions per position. Assembly accession numbers are indicated in brackets.

The phylogenomic analysis revealed similar results like the 16S rRNA gene analysis. Strain IGB 4-14^T^ formed a branch with *F. lacus* within a joint cluster with *F. piscinae, Flavobacterium orientale* and *Flavobacterium filum* (Figure 2). Overall genome relatedness indices substantiated the novel species position of strain IGB 4-14^T^ (Table 1). The dDDH of closely related species resulted in values far below the species boundary of 70% [53]. Accordingly, the ANI and AAI values were below the recommended species cut-off level of 95% [55,56].

In the summary of the results, it becomes clear that strain IGB 4-14^T^ deserves its own species position from a phylogenetic point of view.

### 3.2. Phenotypic Analysis

Strain IGB-14^T^ was comprehensively characterized by a morphological, physiological and chemotaxonomic analysis. The features characterizing the strain and thus the proposed novel species in detail are listed in the protologue (see Section 5). This section focuses on characteristics that allow the strain to be distinguished from the type strain *F. lacus* NBRC 109715^T^, which possess the highest phylogenetic relationship.

The fatty acid profile of strain IGB 4-14^T^ is similar to the type strain of the related species *F. lacus* (Table 2). Both strains display fatty acids iso-C_15:0_ and iso-C_15:1_ G as the major components. Minor amounts were detected for iso-C_15:0_ 3-OH, iso-C_17:0_ 3-OH and iso-C_17:1_ *w9c*, with the proportions differing significantly. Qualitative differences were only found for fatty acids, which occurred in traces, so that a distinction between both strains was mainly based on quantitative differences. For type strains of species clustering in the phylogenomic analysis with the investigated strains, similar fatty acid compositions were described, especially with regard to the dominant acids [57,58]. The profiles match the general description of the genus *Flavobacterium* [18]. Furthermore, the strain IGB 4-14^T^ can be clearly distinguished from the related species *F. lacus* due to several differentiating physiological characteristics (Table 3). These results provide phenotypic support for the separate species status already demonstrated in the phylogenetic analysis. Therefore, the assignment of the investigated isolate to a novel species, *Flavobacterium azooxidireducens* sp. nov., is proposed.

### 3.3. Genome Inventory with Regard to the N-Oxide Reduction

The genome of strain IGB 4-14^T^ does not contain genes coding for NO_3_^−^, NO_2_^−^ and NO reductases. Only the gene of the sec-dependent Clade II NosZ (locus_tag M0M57_RS11105), followed by genes of various accessory proteins of the NosZ, could be detected. Thus, this bacterium possesses only genes for N_2_O reduction, and therefore has the potential to be an important sink for N_2_O without the capabilities to act as an N_2_O source.

**Figure 3:**
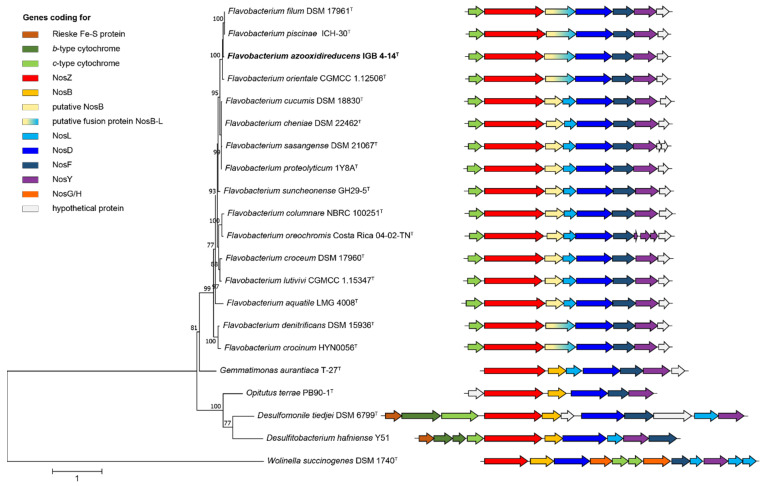
Maximum-Likelihood based phylogeny of NosZ amino-acid sequences showing the position of strain IGB 4-14^T^ among a selection of type strains of *Flavobacterium* species and reference strains according to Sanford, et al. [4]. The scale bar indicates the number of amino acid substitutions per site.

The *nosZ* phylogeny followed the phylogenetic relationship (Figure 3). Strains of the genus *Flavobacterium* constitute a monophyletic branch separate from the reference strains for Clade II NosZ included in the analysis according to Sanford, et al. [4]. Type strains of the species *F. orientale*, *F. filum* and *F. piscinae* formed a cluster with the strain IGB 4-14^T^ similar to the phylogenomic analysis (Figure 2). However, the type strain of *F. lacus*, the species with the closest relationship, does not possess any gene associated with N-transformation processes. These results are consistent with the general view that diversification of *nosZ* occurred predominantly through vertical inheritance, and on a finer scale the distribution of *nosZ* can be uneven, as *nosZ* may be present or absent in the genomes of closely related organisms [2].

The *nosZ* gene of strain IGB 4-14^T^ is followed by genes encoding NosZ asseccory proteins annotated as *nosL* (M0M57_RS11110), *nosD* (M0M57_RS11115), *nosF* (M0M57_RS11120) and *nosY* (M0M57_RS11125). An identical *nos* gene cluster was found for the closely related species, *F. denitrificans* and *F. crocinum* (Figure 3). In contrast, the other flavobacteria involved in the study exhibit two genes at this position between *nosZ* and *nosD*. The first gene is annotated as a hypothetical protein, whereas the second gene is clearly annotated as *nosL*. However, a *nosB* gene typical for a Clade II nos gene cluster was not identified. The *nosB* gene encoding a yet uncharacterized polytopic membrane protein [59] is often surrounded by *nosZ* and *nosD* [4,7]. In *Wollinella succinogenes*, Hein, et al. [60] demonstrated that NosB is essential for N_2_O respiration by characterising a non-polar *nosB* deletion mutant. It is assumed that the protein is involved in electron transport to NosZ, but possibly also in copper management [59]. The latter is derived from the fact that in certain bacteria like *Dyadobacter fermentans* NosB is fused to the copper binding protein NosL [59,60].

It is likely that a fusion protein is also produced by strain IGB 4-14^T^ and flavobacteria, which have an identical *nos* gene cluster (Figure 3). Sequence comparisons of the *nosL* gene of these bacteria with genes encoding the hypothetical protein and NosL of the other flavobacterial group revealed high similarity to the first gene segment and the following segment, respectively. A *nosB* amino-acid sequence comparison of *Gemmatimonas aurantiaca* T-27^T^ with the flavobacterial sequences of the hypothetical protein or the corresponding stretch of the fusion protein revealed identity values between 40 and 46%. Considering conservative substitutions in addition to identical amino-acids, similarity values of around 63% were achieved. In addition, two highly conserved amino-acid stretches were found in the investigated flavobacteria, which were described on the basis of an alignment of NosB homologues by Spanning [59]. In strain IGB 4-14^T^, they were located at positon 76 (LNHYIGM) and 144 (YGHN) of the fusion protein (M0M57_RS11110). These results imply that the hypothetical protein found in some strains is a NosB and the multidomain fusion protein of the other flavobacteria including IGB 4-14^T^ represents a NosB-L that combines the functions of NosB and NosL.

### 3.4. Consumption of N_2_O

Strain IGB 4-14^T^ consumed significant amounts of N_2_O under anoxic conditions (Figure 4a). Following a high consumption rate within the first 14 days, N_2_O consumption plateaued out until almost all N_2_O has been consumed by the end of the experiment. The N_2_O consumption activities were significantly affected by O_2_ levels (Figure 4c). Under microoxic conditions, N_2_O was consumed within the first 6 days. At the same rate, the O_2_ concentration in the test tubes decreased and the CO_2_ production (Figure 4b) increased. With this, the maximum of N_2_O consumption was reached and in the following course of the experiment there were no further changes in the investigated parameters. The average N_2_O consumption rate up to day 6 was 0.090 ± 0.066 µmol d^−1^ and was marginal higher than under anoxic conditions with 0.062 ± 0.022 µmol d^−1^. In contrast, consumption of N_2_O under oxic conditions started around day 6, after most of the O_2_ amount had been consumed. Until then, only low consumption rates of 0.006 ± 0.016 µmol d^−1^ on average were achieved, which increased to 0.054 ± 0.019 µmol d^−1^ in the further course of the experiment. The increase in CO_2_ production correlated negatively with the O_2_ amount in the test tube. When CO_2_ reached its maximum at day 20, N_2_O consumption ceased.

N_2_O reduction by NosZ usually requires anoxic conditions, as NosZ copper centers are susceptible to O_2_ [6]. Nevertheless, N_2_O reduction under oxic and microoxic conditions has been described, for example, for strains of the species *Pseudomonas stutzeri* (Clade I *nosZ*) and *Azospira* sp. (Clade II *nosZ*) [12,61,62,63]. N_2_O reducing bacteria are often facultatively aerobic bacteria isolated from anoxic to microoxic habitats that possess a respiratory chain involved in micro-aerobic respiration. It is assumed that this property gives the cells a certain aerotolerance and protects the N_2_O reductase from inactivation by O_2_ [6]. This could be an explanation for the moderate consumption of N_2_O under microoxic conditions with decreasing O_2_ content in this experiment. Nevertheless, the question arises why N_2_O consumption does not increase after the O_2_ content has been completely reduced. The cessation of CO_2_ formation could indicate exhausted growth. On the other hand, Nos recovery after exposure to O_2_ might be restricted. The latter is considered an important parameter for the selection of strains for N_2_O mitigation in engineered systems [6,12].

Another important result is the lack of CO_2_ formation during anaerobic N_2_O consumption. This indicates that the cells do not grow, which is also reflected in the optical density. During the incubation period, no significant increase in OD was recorded (data not shown). This result implies that N_2_O consumption by strain IGB 4-14^T^ is not coupled to energy conservation for growth, which is in contrast to the common assumption that the reduction of N_2_O is associated with energy conservation but in line with recent studies demonstrating that this is not the case for all microorganisms [10]. *G. aurantiaca* T-27^T^ is one of these organisms that has been extensively studied in terms of physiological properties [64,65]. Although it has been characterized as an obligate aerobic bacterium [66], it is able to reduce N_2_O not only under oxic, but also under microoxic and anoxic conditions when O_2_ was regularly refed [64,65]. The reduction of N_2_O under anoxic conditions apparently acts as an electron sink for metabolism to sustain viability during transient anoxia and is not associated with growth. However, it is not known how widespread this NosZ function is, as it has not yet been tested on a broader diversity of isolates [10]. A further alternative NosZ function is the detoxification of high N_2_O concentrations [2,10,67]. Sullivan, et al. [68] demonstrated the cytotoxic effect of elevated N_2_O concentrations by inactivation of vitamin B_12_, thereby preventing enzyme reactions essential for microbial metabolisms. Thus, in *Paracoccus denitrificans* the B_12_-requiring methionine synthase MetH was inhibited, and the bacterium switched to the vitamin B_12_-independent methionine synthase MetE pathway, through the transcriptional modulation of genes controlled by vitamin B_12_ riboswitches. The two synthases occur together in a large number of bacteria, but in some species only one is present but not the other. In the *Bacteroidota* phylum in particular, MetH is the predominant single enzyme [69]. Correspondingly, the strain IGB 4-14^T^ also possesses only the gene encoding the B_12_-depent synthase MetH (locus tag M0M57_09420), similar to *G. aurantiaca* T-27^T^, which belong to the same clade. Therefore, NosZ can play an important role in the detoxification of high N_2_O concentrations in these organisms. However, the almost complete consumption of N_2_O indicates that strain IGB 4-14^T^ is capable of consuming N_2_O even in the range of natural concentrations in the environment, and along with the demonstrated absence of other denitrification associated enzymes (see above), could be a sink without contributing to N_2_O formation.

## 4. Protologue—Description of *Flavobacterium azooxidireducens* sp. nov.

*Flavobacterium azooxidireducens* (a.zo.o.xi.di.re.du’cens. N.L. neut. n. *azooxidum*, dinitrogen oxide; L. pres. part. *reducens*, reducing from L. v. *reduco*, reduce, bring back to a condition; N.L. part. adj. *azooxidireducens,* reducing nitrous oxide).

Cells are Gram-stain-negative, nonmotile rods, which are approximately 0.3–0.6 µm in diameter and 1.0–1.9 µm long. A capsule is present. Colonies are deep yellow, convex, circular, 1–4 mm in diameter with entire margins. Growth occurs at 4 to 30 °C (optimum 20–25 °C). No growth is observed at 37 °C. It is able to grow on TSA, Reasoner’s 2A agar and nutrient agar. Flexirubin-type pigments are produced, and Congo red is absorbed by colonies. Catalase and oxidase activities are present. Hydrolyses gelatin but not tyrosine, casein, aesculin, starch and DNA. Nitrate is not reduced to nitrite. Arginine dihydrolase, lysine and ornithin decarboxylase, tryptophan deaminase and *β*-galactosidase is not present. Indole and acetoin production is negative.

In the Biolog GEN III Microplate assay following substrates are oxidized: l-arginine, l-aspartic acid, acetoacetic acid, acetic acid, citric acid, dextrin, d-fructose-6-PO_4_, α-d-glucose, d-galactose, 3-methyl glucose, l-glutamic acid, d-galacturonic acid, l-galactonic acid lactone, d-glucuronic acid, glucuronamide, l-histidine, d-lactic acid methyl ester, l-lactic acid, d-maltose, *n*-acetyl-β-d-mannosamine, glycyl-l-proline, *p*-hydroxy-phenylacetic acid, propionic acid, and l-serine. Weak reactions are observed for d-arabitol and d-mannose. No reactions are obtained for d-aspartic acid, l-alanine, γ-amino-butryric acid, α-hydroxy butyric acid, β-hydroxy-d,l butyric acid, α-keto-butyric acid, d-cellobiose, d-fructose, d- and l-fucose, formic acid, gentiobiose, β-methyl-d-glucoside, *n*-acetyl-d-glucosamine, *n*-acetyl-d-galactosamine, glycerol, d-glucose-6-PO_4_, d-gluconic acid, α-keto-glutaric acid, inosine, myo-inositol, α-d-lactose, d-melibiose, d-mannitol, mucic acid, d-malic acid, l-malic acid, *n*-acetyl neuraminic acid, l-pyroglutamic acid, pectin, methyl pyruvate, quinic acid, d-raffinose, l-rhamnose, sucrose, stachyose, d-salicin, d-sorbitol, d-serine, d-saccharic acid, bromo-succinic acid, d-trehalose, d-turanose, and tween 40. The strain is able to grow in the presence of 1% sodium lactate, d-serine, troleandomycin, rifamycin SV, minocycline, lincomycin, vancomycin, nalidixic acid, lithium chloride, potassium tellurite, aztreonam, sodium butyrate and tetrazolium violet. It did not tolerate fusidic acid, guanidine HCl, niaproof 4, tetrazolium blue, and sodium bromate. The strain tolerates pH 6 and a NaCl concentration of 1%, but not pH 5 and 4% NaCl. In the API 20NE test system, d-glucose, d-maltose, potassium gluconate are assimilated, but not l-arabinose, d-mannose, *n*-acetyl-glucosamine, potassium gluconate, capric acid, adipic acid, malic acid, trisodium citrate, and phenylacetic acid.

The predominant fatty acids are iso-C_15:0_ and iso-C_15:1_ G. Minor amounts were detected for iso-C_15:0_ 3-OH, iso-C_17:1_ *w9c*, iso-C_17:0_ 3-OH, and anteiso-C_15:0_.

The type strain IGB 4-14^T^ (LMG 29709^T^ = DSM 103580^T^) was isolated from a mesocosm experiment with decomposing leaf litter of *Phragmites australis* (Cav.) in artificial fen water enriched with fresh detritus originating from a rewetted fen (Germany, 53°52′33.4′′ N, 12°53′20.7′′ E). The DNA G+C content of the type strain is 33.8%. The genome sequence accession number is CP096205 and the 16S rRNA gene accession number is LT598610.

## 5. Conclusions

In this study, a bacterium associated with decomposing leaf litter of *Phragmites australis* (Cav.) was clearly differentiated from related *Flavobacterium* species in a polyphasic approach applying phylogenomics and phenotypic analysis. Based on these data, a novel species *Flavobacterium azooxidireducens* sp. nov. with the type strain IGB 4-14^T^ was proposed. The type strain is a non-denitrifying bacterium that was able to consume significant amounts of N_2_O under anoxic conditions by a N_2_O reductase of the Clade II. Due to the absence of other enzymes associated with denitrification, it has the potential to be a sink without contributing to N_2_O formation. The N_2_O consumption served as an electron sink for metabolism and/or to detoxify high N_2_O concentrations. It shows a high similarity in *nos* gene cluster composition and amino acid similarity of the *nosZ* gene with different type strains of the genus, so it can be assumed that the N_2_O reductase in these strains might also underlie this function and is typical for the genus *Flavobacterium*. However, in contrast to the NosZ function for energy conservation through anaerobic respiration, the importance and distribution of these alternative NosZ functions in the environment is not known and needs further investigation.

## Figures and Tables

**Table 1:** Overall genome relatedness indices and 16S rRNA gene similarities calculated for the strain *F. azooxidireducens* IGB 4-14^T^ and closely related *Flavobacterium* species.

Strain IGB 4-14^T^ vs. Species	16S rRNA Gene Similarity	dDDH Values (%)	ANI (%) ^2^	AAI (%)
*F. lacus* CGMCC 1.12504^T^ (NP180^T^) ^1^	97.29	32.2	86.68	89.78
*F. piscinae* ICH-30^T^	96.92	27.8	84.01	87.92
*F. orientale* CGMCC 1.12506^T^ (SP3^T^) ^1^	96.80	24.9	80.31	81.02
*F. filum* DSM 17961^T^ (EMB34^T^) ^1^	94.57	24.1	80.52	83.20

^1^ Strain number for analysis of 16S rRNA gene similarity if different from the genome analysis. ^2^ Coverage 37–48%.

**Table 2:** Whole cell fatty acid compositions of strain *F. azooxidireducens* IGB 4-14^T^ and the type strain *F. lacus* NBRC 109715^T^.

Fatty Acid ^1^/Strain	IGB 4-14^T^	NBRC 109715^T^
**Saturated**		
iso-C_13:0_	1.32	Tr
anteiso-C_13:0_	Tr	ND
C_15:0_	Tr	2.4
iso-C_15:0_	44.18	36.11
anteiso-C_15:0_	2.36	1.1
iso-C_15:0_ 3-OH	8.57	6.2
iso-C_17:0_ 3-OH	5.30	8.49
**Unsaturated**		
iso-C_15:1_ G	17.65	21.58
C_15:1_ *w6c*	Tr	2.61
iso-C_17:1_ *w9c*	5.63	9.88
anteiso-C_17:1_ *w9c*	Tr	ND
C_17:1_ *w6c*	ND	Tr
C_17:1_ *w8c*	ND	Tr
**Summed features ^2^**		
Feature 1	1.52	ND
Feature 3	1.92	1.71
Feature 4	Tr	1.68

TR, trace (<1.0%). ND, not detected. ^1^ Fatty acids amounting to less than 1.0% of the total fatty acids in both strains are not mentioned in the table: C_14:0_, iso-C_14:0_, iso-C_14:0_ E, C_15:0_ 2-OH, C_16:0_, iso-C_16:0_, iso-C_16:0_ 3-OH, iso-C_17:0_, C_17:0_ 2-OH, anteiso-C_15:1_ A, iso-C_16:1_ H, and C_18:1_ *w5c*. ^2^ Summed feature 1: iso-C_15:0_ H/C_13:0_ 3-OH; summed feature 3: C_16:1_ ω7*c*/iso-C_15:1_ 2-OH; summed feature 4: iso-C_17:1_ I/anteiso-C_17:1_ B.

**Table 3:** Physiological characteristics distinguishing strain *F. azooxidireducens* IGB 4-14^T^ and the type strain *F. lacus* NBRC 109715^T^.

Characteristic	IGB 4-14^T^	NBRC 109715^T^
Flexirubin-type pigments	+	-
absorption of Congo red	+	-
**Oxidation (GEN III) of:**		
*N*-Acetyl-β-D-Mannosamine	+	-
D-Galactose	+	-
3-Methyl Glucose	+	-
D-Fructose-6-PO_4_	+	-
L-Arginine	+	-
L-Histidine	+	-
Pectin	-	+
D-Galacturonic Acid	+	-
L-Galactonic Acid Lactone	+	-
D-Glucuronic Acid	+	-
Glucuronamide	+	-
Mucic Acid	-	+
*p*-Hydroxy-Phenylacetic Acid	+	-
L-Lactic Acid	+	-
D-Malic Acid	-	+
L-Malic Acid	-	+
Propionic Acid	+	-

## Data Availability

The GenBank/EMBL/DDBJ accession numbers for the 16S rRNA gene sequences and the IGS spacer region of type-strain *Flavobacterium azooxidireducens* IGB 4-14^T^ are LT598610 and LT616948, respectively. The genome sequence data for the type-strain are available under the GenBank/EMBL/DDBJ accession number CP096205. It has been deposited in the German Collection of Microorganisms and Cell Cultures GmbH (=DSM 103580^T^) and in BCCM/LMG Bacteria Collection in Belgian (=LMG 29709^T^).
